# The role of education in health policy reform outcomes: evidence from Japan

**DOI:** 10.1007/s10198-023-01568-9

**Published:** 2023-02-08

**Authors:** Masato Oikawa

**Affiliations:** 1 School of Education, Waseda University, Tokyo, Japan; 2 Waseda Institute of Social & Human Capital Studies (WISH), Tokyo, Japan

**Keywords:** Health policy, Health checkup, Health information, Education, Obesity, BMI, Health investment, Difference-in-differences (DID) estimation, I12, I18, I20

## Abstract

This study analyzes the role of education in the outcomes of the reform of the Japanese annual health checkup program. In April 2008, the annual checkup was redesigned to address concerns about metabolic syndrome. As the checkup is mandatory only for salaried workers, their participation rate is significantly higher than other workers; thus, they were most affected by the reform. Using institutional information, a difference-in-differences estimation was conducted with salaried workers as the treatment group and self-employed workers as the control group. We found that the reform caused significant changes in health behaviors and outcomes only among university graduates who were at a relatively high risk of metabolic syndrome. This highly educated group increased their physical activity, brought energy intake close to an ideal level, and achieved significant weight loss and BMI reduction to levels that minimize all-cause mortality among middle-aged Japanese. A secondary analysis implies that the difference in cognitive functioning test scores may be a critical factor in explaining the heterogeneous responses to the reform, suggesting that thoroughly well-articulated recommendations for healthy behaviors are needed in order to improve reform uptake.

## Introduction

Economists are increasingly interested in the effect of education on economic and non-economic outcomes, especially the relationship between education and health. Since Grossman [15], the relationship has been actively investigated both theoretically and empirically, with the literature reviewed by Grossman [16] and updated by Eide and Showalter [11] and Grossman [17]. While most empirical studies to date focus on the causal effect of education on health, less attention has been paid to the specific underlying mechanism, which Grossman [17] pointed out is an area of future work that has important implications for effective health policy implementation.

One possible mechanism is that highly educated individuals might respond to new information and change their behaviors more quickly. In other words, individuals for whom education has been a successful endeavor may be more “teachable.” This possibility has received support from a range of studies on the uptake of newly-approved drugs [29]; for example, a national information campaign on the HIV/AIDS epidemic in Uganda [9], a medical research publication which demonstrated that the risks of attempting a vaginal birth after having a previous C-section birth are higher than previously thought [35], and several studies since the 1950s of the growing awareness of the negative effects of smoking, as reported in the popular press [10] and the 1964 U.S. Surgeon General’s Report on Smoking and Health [1, 10].

A detailed study of the specific mechanisms behind heterogeneous responses by level of education can help policymakers consider new effective policies. In some of the abovementioned studies, however, the interval between the receipt of information and subsequent behavior is long, which makes it difficult to determine the specific mechanism in detail. For example, there are at least two possible paths by which level of education could lead to a heterogeneous response to new health information, each with very different implications for policy. One is that more highly educated individuals might have better access to new health information, but another possibility is that they might respond more quickly or efficiently to new health information even when access is equal. If highly educated individuals have better access to new health information, it is important to find ways to disseminate this information widely. If access to information is not the problem, however, then it is important to devise more effective ways to encourage more widespread behavioral change.^1^ This might include, for example, health campaigns that are much easier to understand, with practical recommendations, specific implications, and concrete examples. This study uses a change in the health checkup system in Japan aimed at reducing obesity as the clear mechanism underlying the causal relationship between education and health. Because all workers were aware of the reform and it did not alter participation in the checkup itself, this study discusses the latter possible path by which differences in education lead to heterogeneous responses to policy reform.

Another limitation of the extant literature is that most studies are not quasi-experimental in that they compare changes in outcome variables among subjects who are highly or poorly educated without defining clear treatment and control groups. Therefore, while the heterogeneous responses to new information received by these two groups may seem intuitive, the studies cannot claim direct causal effects. By contrast, we use an institutional setting, worker type, to identify the policy effects by comparing the changes in health outcomes and behaviors between two groups with higher and lower proportions of members affected by the policy reform.

While economists have studied the effects of an exogenous variation in assimilating health-related information through various interventions, including health checkups and screening programs, on health outcomes and behaviors in Austria [18], China [40], Japan [13, 21, 22, 26], Korea [27], and the United States [2, 24, 33] to date, there is no unified view of the effects. Some studies have found evidence for the improvement of health by receiving health information through health checkups, health guidance, and health screenings [e.g., [13, 21, 27]]. Iizuka et al. [21], for example, found health improvement due to diabetes diagnoses among individuals with high health risks. They also found that health improvement is worth the medical spending on other preventive care increased due to the diagnoses. Fukuma et al. [13] found evidence for weight loss, BMI reduction, and waist circumference reduction due to the Specific Health Guidance in Japan one year after screening, but the health improvements were attenuated a few years later.^2^ In contrast, others found no evidence for health improvement [e.g., [2, 18, 24]]. For the effects on health behaviors, there are previous studies that found statistically significant changes in health behaviors towards health improvement [e.g., [26, 33, 40]], while the others found no evidence [e.g., [2, 24, 27]].

One possible reason for the mixed results is the estimation methodology used in previous studies. Most of the studies in the extant literature apply a regression discontinuity design (RDD) with a biomarker threshold for diagnosing a health condition such as high blood pressure [40], diabetes [2, 21, 27], obesity [27], or waist circumference [13]. Since biomarkers are affected by various exogenous factors such as timing, measurements just above and below a threshold are likely to be random and the effect of a diagnosis on subsequent health outcomes and behaviors can be estimated around that threshold. Although this strategy has strong identification power, the estimated effects appear to be highly localized, and it is difficult to interpret insignificant effects.^3^ Further, these studies have utilized a variety of thresholds for the identification strategy, and, moreover, have found heterogeneous effects based on numerous factors including level of income [40], age [18], and level of health risk [21, 27]. Taking this into consideration, as it is likely that these studies investigated different estimands making the estimated results somewhat incomparable, further investigation of the effects of health checkups is necessary to gain consensus.

Additionally, there are few studies of health diagnoses focusing on heterogeneity by level of education. One study by Zhao et al. [40] utilizes an RDD framework to analyze the heterogeneous effects of a hypertension diagnosis on nutrition intake by both education and income, finding that the effect on fat intake is stronger among those with a lower education and higher income, which is inconsistent with the literature on the relation between education and health. One possible reason is that an individual who knows the diagnosis threshold may change their behavior upon learning that their blood pressure rating is just below the threshold because they understand that they are still at risk. Highly educated individuals may be more likely than poorly educated individuals to know the diagnosis threshold. Therefore, this strategy does not seem ideal for analyzing the heterogeneity of effects by level of education,^4^ leaving ample room for more studies of heterogeneous effects of health checkups by level of education.

This study utilizes a reform of the health checkup system in Japan aimed at reducing metabolic syndrome to analyze the heterogeneous responses of health behaviors and outcomes to the policy reform by education level. Japan has one of the most aged populations in the world and faces the problem of extended medical expenditure on lifestyle-related diseases including diabetes and hypertension. The percentage of males who are overweight, a risk factor for lifestyle-related diseases, increased by 32 % in the 20 years to 2017, and the number of people strongly suspected of having diabetes also increased by 45 % in the 20 years to 2016. Therefore, the Japanese government has reformed the health checkup system aimed at reducing the overweight population and eventually the people with lifestyle-related diseases.^5^ Recognizing that the reform did not affect the participation rate in checkups but that the proportion of workers affected by the reform differed exogenously according to their work status, the study uses a difference-in-differences (DID) approach to compare the changes in the outcome variables between two groups with higher and lower proportions of members affected by the policy reform.

The results show that while the DID estimates of weight and body mass index (BMI) are statistically significantly negative for university graduates at higher risk of obesity, there are no significant changes among non-graduates at higher risk of obesity or individuals at relatively low risk of obesity. This highly educated group achieved significant weight loss, reducing BMI to levels that minimize all-cause mortality. Further, among university graduates at high risk of obesity, health behaviors such as physical activity and eating habits also changed. These results suggest that highly educated individuals are more likely to respond to a health checkup diagnosis and/or health guidance to improve their health. A secondary analysis suggests that cognitive functioning may be a key factor explaining this heterogeneity of response, which is consistent with other recent discussions of the role of cognition in the causal relationship between education and health [e.g., [3, 4, 8]].

The remainder of the paper is organized as follows: “Institutional background” explains the institutional setting; “Data” discusses the data and descriptive statistics; “Identification strategy” describes the identification strategy and estimation model; “Estimation results” discusses the estimation results; “Discussion” provides some additional remarks; and “Conclusion” summarizes the study, discusses limitations, and makes suggestions for further research.

## Institutional background

### Japan’s annual health checkup

Since the 1970s, an annual health checkup has been provided as part of Japan’s health promotion policy. The Industrial Safety and Health Act of 1972 requires all employers to provide a health checkup for their employees, who are, in turn, legally obligated to take it. Thus, virtually all salaried workers in Japan undergo a health checkup each year. Although the checkup may include additional tests, a set number are required by law. As such, salaried workers across Japan all receive a uniform minimum-level checkup. In addition to the checkups provided by employers, local governments also provide checkups for residents over 40 who are not salaried workers. Thus, all middle-aged and above Japanese residents have the opportunity to receive an annual health checkup, although it is mandatory for salaried workers and voluntary for others, including the self-employed.

However, in the early 2000s, despite these publicly provided checkups having taken place annually for decades, health had not sufficiently improved. Specifically, according to the mid-term evaluation of the “Health Japan 21” policy aimed at reducing the prevalence of lifestyle-related diseases and implemented in 2000 at the turn of the 21st century, the incidence of lifestyle-related health conditions such as diabetes and obesity had increased. As these health conditions comprise a large proportion of public health expenditure, the problems with the health checkups were investigated and summarized in a Council of Governments report.^6^ The first problem identified was that the existing intervention did not work for people who already had a disease. While screening is most effective for people at high risk of a disease but who do not yet have it,^7^ the purpose of the checkups, up to that point, was to select for intervention patients who were already in the early stages of a disease. The second problem with the health checkups was that the intervention was insufficient. While Knowler et al. [28], for example, found that for those at high risk of diabetes, intervention to help change lifestyle habits was more effective in preventing the onset of diabetes than medication, the health checkups merely provided those identified as high risk with general information about the disease and a recommendation to see a doctor. The final problem was that the content of the health checkups, conducted by providers across the country under various local laws, was not unified. Addressing these identified inadequacies of the existing health checkups thus required a reform of the system to provide a more substantial intervention targeting those at high risk of disease and implemented uniformly across all institutions nationwide. This new system is also a part of the “Health Japan 21” policy.

### Specific health checkups and specific health guidance

**Fig. 1:**
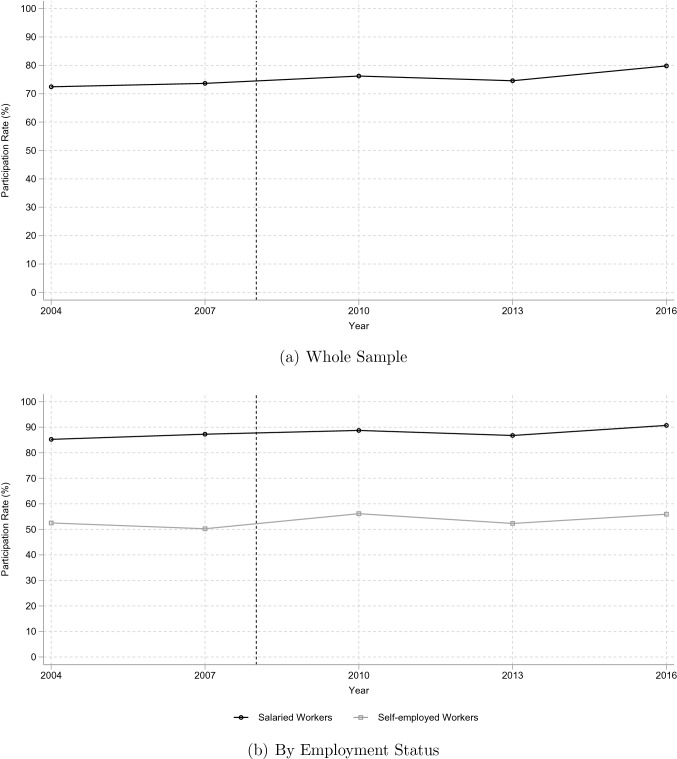
Participation rate in health checkups of the middle-aged. Source: Comprehensive Survey of Living Conditions. Participation rate is calculated for males aged 50–62

In April 2008, a new health checkup system, Specific Health Checkups and Specific Health Guidance, was introduced, aimed at preventing lifestyle-related diseases by providing participants with objective assessments of their health risks and specific guidance from health professionals. This new health checkup system now focuses on metabolic syndrome, a condition represented by a confluence of biomarkers including excess body fat, high blood pressure, and high blood sugar which, together, identify people at high risk of lifestyle-related diseases. The policy reform was introduced uniformly for the target population of individuals covered by public health insurance and their dependents aged between 40 and 74. Since Japan has a universal health care insurance system, this target population covers almost all residents of the country. Importantly for this study, the reform did not alter participation in health checkups. As seen in Panel (a) of Fig. 1, there is no surge in the checkup participation rate of the middle-aged around the policy reform.

The current system is divided into two parts: a health checkup to screen for participants at high risk of metabolic syndrome, followed by face-to-face guidance by a doctor, public nurse, or dietitian aimed at prevention by changing lifestyle habits. The content of the checkup is based on medical and scientific evidence for identifying metabolic syndrome, and includes body measurements, blood tests, and questionnaires about such topics as smoking and medical histories. As excess body fat is a marker of metabolic syndrome, a measure of abdominal girth was added to the new system to estimate the amount of visceral fat. Based on the results of the health checkup, an objective assessment of metabolic syndrome risk is determined.

Participants then receive health guidance specifically tailored to their physical condition. Those at high risk are given health guidance about their lifestyle habits, aimed at informing participants of the benefits and risks of their lifestyle habits and providing support to change behavior. There are two types of health guidance: Motivational support for participants who have risk factors of metabolic syndrome, but the number of risk factors is relatively low; and active support for participants who have more risk factors of metabolic syndrome. Active support guidance is a support program with higher intensity than motivational support guidance. In both types of guidance, the participants are required to set their weight loss goals and make a lifestyle-change plan to achieve them.^8^

In short, the policy reform provides checkup participants with objective knowledge of the risks associated with their health condition and specific information about the benefits and risks of their health behaviors.^9^

## Data

The main dataset used in this study is the Japanese Study of Aging and Retirement (JSTAR), a biennial panel survey of Japanese people aged over 50 which is a sister dataset of the U.S. Health and Retirement Study (HRS), the English Longitudinal Survey on Ageing (ELSA), and the Survey on Health, Ageing, and Retirement in Europe (SHARE). The first wave of the JSTAR was conducted in 2007 in five cities in Japan. Although other sample cities were added in later waves, this study employs only the five original cities in order to obtain data both before and after the health policy reform of 2008. The JSTAR includes comprehensive information on demographics, labor force status, economic variables, health investment behaviors, and health outcomes to analyze the impact of the checkup system reform on health outcomes and behaviors.^10^ The purpose of the policy reform was to reduce the number of people at high risk of metabolic syndrome, and one solution is weight loss. This is captured in this study through measures of weight and body mass index (BMI), or body weight adjusted by height, a common measure of obesity.

In addition to the JSTAR, the Comprehensive Survey of Living Conditions, the Longitudinal Survey of Middle-aged and Elderly Persons, and the General Survey on Working Conditions are also used in this study to interpret and provide further context for the main estimation results.

## Identification strategy

This study utilizes heterogeneity in the health checkup participation rate to identify the effects of the policy reform on health outcomes and behaviors. As noted above, the new checkup system was implemented uniformly, making it difficult to assign participants to treatment and control groups on that basis, so a different institutional setting was used based on employment status. As explained above, the annual health checkup has always been mandatory for salaried workers, so their participation rate is higher than others. Panel (b) of Fig. 1 shows the participation rate of middle-aged workers according to employment status and indicates that the participation rate of salaried workers is about 90% and constant before and after the policy reform. Therefore, about 90% of salaried workers were affected by the policy reform but their participation in health checkups did not change. On the other hand, the participation rate of self-employed workers is substantially lower, at about 50%, and also shows no significant change in participation around the time of the policy reform. We can, therefore, conclude that about 50% of self-employed workers were affected by the reform, but their participation in health checkups did not change.

We use this stable difference in the proportion of workers affected by the policy reform as the identification strategy, applying a difference-in-difference (DID) framework to compare the before-after change in health outcomes between the salaried worker, i.e., the treatment group with a higher proportion of members affected by the reform, and the self-employed worker, that is, the control group with a lower proportion of members affected.^11^,^12^ Within this DID approach, the estimated effects are deducted by the difference in the participation rate between salaried and self-employed workers, so if the signs of both groups are the same, the DID estimate indicates the lower bound of the magnitude of the effect in absolute value. The interpretation of the DID estimate is discussed further in “Discussion”.

In order to ensure the validity of the DID approach, a number of assumptions must hold: First, it is important for the identification strategy that the relative participation in health checkups does not change between the treatment and control groups. As the checkup has always been mandatory for salaried workers, one would not expect their participation to change, and this has been confirmed above. For self-employed workers, however, the checkup is voluntary and so it is conceivable that their participation in the improved checkups might increase. If their participation rate were to rise to something approximating that of salaried workers, the shrinking difference in participation rates would reduce the effectiveness of the DID approach. However, as discussed above, the participation rate did not change significantly after policy reform for either salaried or self-employed workers (Panel (b) of Fig. 1).

Second, an important assumption for the internal validity of DID is the common trend assumption; namely, that counterfactual changes in outcomes among salaried and self-employed workers must be the same if policy reform is considered to not have occurred. This allows us to measure the effect of the reform. The typical means of testing for this is to check the trends in target outcomes before the reform, but as our dataset includes only one period before the policy reform, we must test this via other means. First, as the JSTAR asks respondents to self-report the change in their health from one year before the survey to the survey date, we use this information to assess whether the trends of salaried and self-employed workers are heterogeneous. Second, as the dataset is comprised of longitudinal data that includes rich information about demographic, economic, and health related variables, we can control for individual observable characteristics related to health such as age and economic condition, as well as time-invariant individual heterogeneity. Third, in order to provide further evidence of a common trend, a placebo regression using a health variable less related to the newly introduced system was estimated. While the details of these validity checks are discussed below, at this point we can state that there is no evidence implying the violation of the common trend assumption.

### Estimation equation

Controlling for any potential bias caused by heterogeneity in the trends of salaried and self-employed workers, the estimation equation is as follows:

1 $$\begin{aligned} y_{it}= & {} \beta _{0} + \beta _{1} SalariedWork07_{i} + \beta _{2} After_{t} \nonumber \\&+ \beta _{3} SalariedWork07_{i} \cdot After_{t} + x'_{it} \delta + \theta _{i} + \epsilon _{it} \end{aligned}$$

where *i* and *t* are indices of individual and time. The dependent variable $y_{it}$ represents health outcomes such as weight and BMI, and health investment behaviors such as physical activity and eating habits. $SalariedWork07_{i}$ takes one if the respondent was a salaried worker in 2007 before the policy reform, while $After_{t}$ takes one after the policy reform. The vector $x_{it}$ is a set of control variables that includes age dummy variables, marital status, number of children, household income, house ownership, hours of work, stress condition at the workplace, occupation variables,^13^ and prefecture-level macroeconomic variables. Parameter $\theta _{i}$ captures the unobserved individual fixed effects and parameter $\epsilon _{it}$ is an unobserved error term. In Eq. (1), parameter $\beta _{3}$ corresponds to the DID estimate and is the parameter of interest in this study. This captures the difference in the change in the outcome variable between salaried and self-employed workers in 2007. Equation (1) is estimated for both university graduates and non-graduates and the DID estimates are compared in order to discover the heterogeneous effects of education on the outcomes of policy reform.

### Verification of common trend assumption

As discussed above, any causal interpretation of the DID estimate requires the common trend assumption to hold. Although our dataset includes only one period before the reform, we can use the 2007 self-reported change in health from the previous year to check for any heterogeneity in the trends. We present the self-reported change in health from 2006 to 2007 by level of education and employment status in Table 1.^14^ We see that the patterns of the self-reported change in health are not statistically significantly different between salaried and self-employed workers for both university graduates and non-graduates. Most of the males in our sample answered that their health conditions were the same as the previous year’s. This suggests that the common trend assumption between salaried and self-employed workers is not violated, at least by what can be captured by self-reported changes in health.

Next, as an additional measure to control for any heterogeneity in the trends between salaried and self-employed workers, observable characteristics were added to the model and a fixed effects estimation was conducted using the panel structure of the JSTAR. In addition to the demographic variables of age, marital status, and number of children, household income and home ownership economic variables were included as control variables because previous studies have shown a relationship between health and economic conditions [e.g., [6, 7, 38]]. Further, because the analysis sample includes the JSTAR data during the financial crisis of 2008 which may have affected workplace and regional economic conditions heterogeneously, workplace-related variables (hours worked, physical stress at workplace, job stress at workplace, occupation category dummy variables), and time-variant regional characteristics (prefecture-level GDP and per capita income) were also included as controls. Additionally, although the accumulation of health stock until middle age and health preferences could also cause heterogeneity in the trends, the fixed effects estimation controls for such time-invariant unobserved individual heterogeneity.

As a final measure to ensure that the common trend assumption is satisfied, placebo regressions were run using mental health measurements and height as the dependent variables. As alluded to above, one concern regarding the validity of the common trend assumption is the financial crisis of 2008. The analysis sample includes the JSTAR data during the financial crisis which could change individual behaviors heterogeneously, resulting in heterogeneous changes in health conditions, including body weight. Therefore, heterogeneous behavior changes due to the financial crisis may cause some violations of the common trend assumption. We run the same estimation model with the measurements of mental health conditions, which have been said to be associated with economic recessions in previous studies, as dependent variables to discuss whether the financial crisis heterogeneously affected individuals.

Height is also used as a dependent variable for the placebo regression as it is a component used to calculate BMI and it is known to decline after middle-age. For Japanese middle-aged males, height loss begins at age 41, and annual height loss is estimated between − 0.01 and − 0.17 cm [34].^15^ Suppose that height declines only among self-employed workers and body weight remains the same among both salaried and self-employed workers. This could produce results implying BMI decline due to the policy revision even without weight loss.^16^ The financial crisis could change individual behaviors heterogeneously, resulting in heterogeneous height loss. In addition, heterogeneous height loss could also occur due to a difference in health decline between salaried and self-employed workers, because significant height loss after middle age is used as a proxy for health decline [12, 19]. To eliminate these possibilities, we also implement the placebo regression using height as the dependent variable.

### Analysis sample restrictions

For the estimation, the analysis sample was first restricted to males aged between 50 and 62 because the identification strategy requires workers and in Japan, and males are more likely to be working. Next, although the newly introduced health checkup system was made available to everyone aged 40 and over, a minimum age of 50 was chosen for this study because the JSTAR data included only those aged 50 and over. Meanwhile, the maximum age was restricted to 62 in order to eliminate workers who were eligible to retire with a pension from the sample, as previous studies have found that this affects health conditions and behaviors [e.g., [25, 30–32, 41]]. In the first wave of the JSTAR, 62 is the age that people are eligible for a full pension.^17^

Next, the sample was divided into two groups according to their risk of metabolic syndrome before the policy reform because, as mentioned above, individuals at a high risk received objective results of the checkup and guidance from a health expert. Moreover, low risk individuals do not need to change their behaviors because they are already healthy. For these reasons, there is a possibility that the reform effects might be heterogeneous according to the individual’s potential risk of metabolic syndrome.

**Fig. 2:**
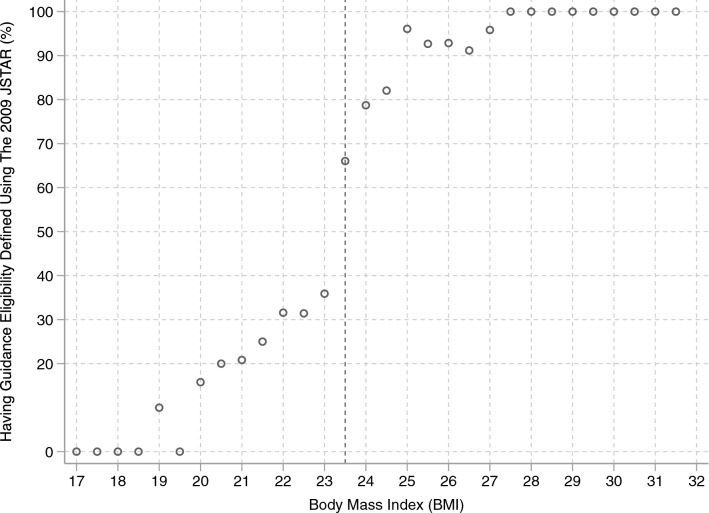
Relationship between BMI and eligibility for health guidance (2009). Source: JSTAR 2009. Sample: Males aged over 50

BMI was used as the criterion to divide the sample by health condition before the reform. As explained above, a range of measurements are used to evaluate the risk of metabolic syndrome, but as the 2007 JSTAR includes only BMI information, it is difficult to construct a full picture of the pre-reform risk of metabolic syndrome from that alone. Fortunately, however, the 2009 JSTAR also includes information on girth of abdomen and blood pressure that can be used to gauge an individual’s eligibility for guidance.^18^ A plot of these two pieces of information, i.e., eligibility for guidance and BMI, was used to determine the BMI criterion for dividing the sample into high and low risk. From Fig. 2, we see that the probability of receiving guidance increases as BMI rises, but there is a jump of about 30 percentage points when BMI reaches 23.5, from 40 % at BMI = 23–70 % at BMI = 23.5. Thus, BMI greater than 23.5 became the criterion for defining the high-risk portion of the sample, with others at low-risk of metabolic syndrome. Because those at high risk have an incentive to change their behavior and reduce their obesity, the policy reform effects should be stronger for these individuals; therefore, the analysis sample was further restricted to them.

## Estimation results

### Descriptive statistics

**Table 1:** Differences in characteristics between salaried and self-employed workers by education before the policy reform

	University Graduates			University Non-Graduates		
	(1)	(2)	(3)	(4)	(5)	(6)
	Salaried workers	Self-employed workers	Diff.	Salaried workers	Self-employed workers	Diff.
Demographic variables						
Age	55.27	56.00	− 0.73	56.62	56.86	− 0.24
= 1 if married	0.92	0.85	0.06	0.86	0.92	− 0.06
Number of children	1.80	1.94	− 0.15	1.93	2.11	− 0.18
Economic variables						
Household income(10k JPY)	900.07	860.44	39.63	601.42	592.34	9.08
House ownership	0.79	0.74	0.05	0.76	0.84	− 0.08
Workplace environments						
Hours worked	47.67	43.97	3.70	45.15	49.66	− 4.51***
= 1 if physically stressed	0.20	0.18	0.02	0.42	0.60	− 0.18***
= 1 if feeling pressed for time	0.54	0.41	0.12	0.42	0.51	− 0.10
Body measurements						
BMI	25.78	25.93	− 0.16	25.68	25.66	0.02
Weight (kg)	74.41	72.59	1.82	72.16	71.67	0.49
Height (m)	1.70	1.67	0.03**	1.68	1.67	0.01
Preference for health						
Be currently interested in own health	0.89	0.85	0.04	0.88	0.81	0.08
Have confidence for own health 3 years later	0.35	0.35	− 0.00	0.36	0.31	0.05
Self-reported change in health						
How is your current health compared to one year ago?						
Better	0.05	0.03	0.02	0.04	0.05	− 0.01
Same	0.86	0.82	0.03	0.87	0.81	0.06
Worse	0.10	0.15	− 0.05	0.09	0.14	− 0.05

Source: JSTAR 2007

*$$p<0.1$$, **$$p<0.05$$, ***$$p<0.01$$

Values are calculated for males aged 50–62 whose BMI before the policy reform was 23.5 or greater

Before presenting the estimation results, this section discusses relevant descriptive statistics of the sample. First, looking at males between 50 and 62 who were at high risk of metabolic syndrome before the policy reform, Table 1 shows the average values of observable characteristics and the difference in those characteristics between salaried and self-employed workers by level of education in 2007. According to Table 1, after conditioning for health risk before the reform, most of the characteristics are not statistically different between salaried and self-employed workers, regardless of whether they were university graduates or not (Columns (3) and (6)). Average BMI is similar for all groups, ranging from 25.66 to 25.93. Therefore, while we were concerned that if more obese individuals decreased their weight more, this would lead to heterogeneity in the effects of the policy reform on BMI, this concern appears unfounded because BMI is similar across groups. We also notice that salaried workers with university degrees appear to be taller, but the difference is small (about 1.8 %). Next, looking at workplace conditions, we notice that among those with lower education, the self-employed work about 10% more hours than salaried workers and are more physically stressed. This means that salaried workers enjoy more leisure time, which can be used to invest in positive health behaviors, such as exercise. Additionally, the difference in physical effort required at the workplace may affect weight loss. Therefore, because these two differences can cause the estimates to be biased, it is important to control for these workplace conditions, especially for non-graduates.

**Fig. 3:**
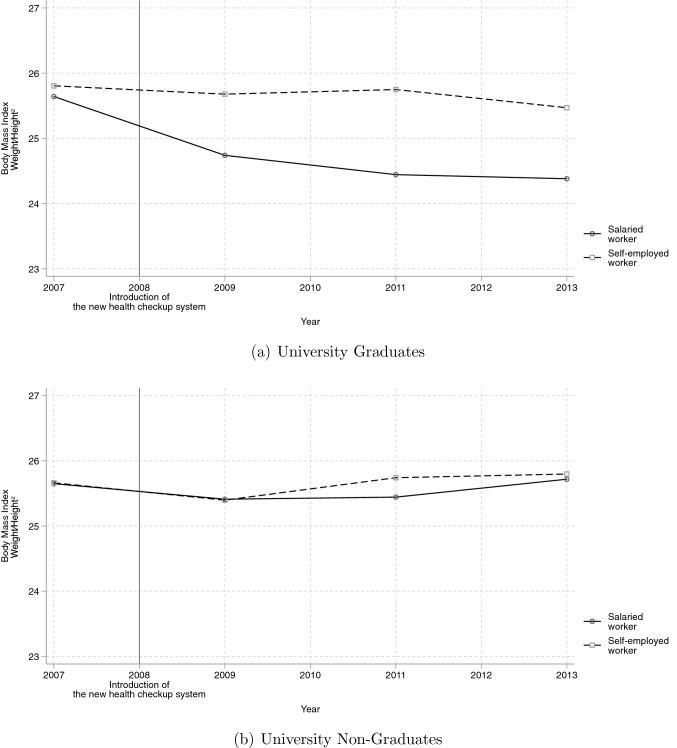
Changes in BMI among individuals at high pre-reform risk by employment status and level of education. Source: JSTAR. Sample: Males aged 50–62 whose BMI before the policy reform was greater or equal to 23.5

Before presenting the estimation results for Eq. (1), we first discuss the DID estimates via Fig. 3. Figure 3 summarizes the changes in average BMI among individuals at high pre-reform risk of metabolic syndrome by their pre-reform employment status and level of education. Panels (a) and (b) show the changes for university graduates and non-graduates, recalling that average BMI was approximately the same for all groups in 2007. From Fig. 3, we see that for salaried workers with university degrees, average BMI falls below 25 in 2009 and remains at that level throughout (solid line in Panel (a)), while all other groups show no decrease in average BMI (dashed line in Panel (a) and lines in Panel (b)). This suggests that the goal of the reform, that is, obesity reduction, occurred only for salaried workers, for whom a larger proportion of members were affected by the reform, and only if they had a higher level of education.

While this result does not completely rule out the possibility of bias arising from the common trend assumption failing to hold, in the estimations discussed below, an attempt is made to minimize any such bias by controlling for observable characteristics and time-invariant individual heterogeneity.

### Effect on health outcomes

**Table 2:** Effects of policy reform on body measurements by education (males aged 50–62 with high pre-obesity risk)

	University graduates			University non-graduates		
	(1)	(2)	(3)	(4)	(5)	(6)
	BMI	Weight (kg)	Height (m)	BMI	Weight (kg)	Height (m)
Salaried before policy reform $\times$ after	− 1.065***	− 2.987***	0.002	− 0.021	0.166	0.003
	(0.321)	(1.071)	(0.004)	(0.287)	(0.740)	(0.002)
Number of observations	202	202	202	480	480	480
Control variables	Yes	Yes	Yes	Yes	Yes	Yes
Individual FE	Yes	Yes	Yes	Yes	Yes	Yes
Mean before the reform						
Salaried	25.78	74.41	1.70	25.68	72.16	1.68
Self-employed	25.93	72.59	1.67	25.66	71.67	1.67

*$$p<0.1$$, **$$p<0.05$$, ***$$p<0.01$$

All specifications are estimated using an FE model. Clustered robust standard errors are in parentheses

Included are age and marriage dummy variables, number of children, income, home ownership, hours worked, physical stress at the workplace, job stress at the workplace, current occupation dummy variables, cross terms of the occupation categories at 1st wave and survey year dummy variables, and prefecture level macroeconomic variables, such as GDP and income per capita

This section discusses the estimation results for the effect of the reform on the measurements of BMI, weight, and height. Table 2 shows the estimation results for those at higher risk of obesity before the policy reform by level of education, with Columns (1), (2), and (3) showing university graduates and Columns (4), (5), and (6) showing non-graduates. Fixed effects (FE) estimation was used in all specifications to control for individual time-invariant heterogeneity, and the DID estimate, $\beta _{3}$ of Eq. (1), is reported.

According to Table 2, the DID estimate of BMI for university graduates at higher risk of obesity before the policy reform is − 1.065 and statistically significant at the 1% level after controlling for observable characteristics and unobserved time-invariant heterogeneity (Column (1)). Since average BMI for salaried workers with university degrees before the policy reform was 25.78, this means that their BMI decreased by about 4.1% to approximately 24.72. A medical study by Tsugane et al. [39] analyzing the relationship between BMI and all-cause mortality for middle aged Japanese finds that the mortality profile for males has a U-shape and bottoms out at a BMI range of 23.0–24.9. Consequently, we can interpret the observed reduction of BMI among university graduates to within this range as an improvement in their health condition. The effect of the policy reform on BMI is non-linear: the higher the pre-reform BMI, the larger the magnitude of BMI reduction due to the policy reform.^19^ As explained in “Institutional background”, the higher the risk of metabolic syndrome, the more intense the health guidance that is implemented. The non-linear effects of the policy reform on pre-reform BMI may reflect the difference in the intensity of health guidance by the risk of metabolic syndrome.

Additionally, among university graduates, we find a statistically significant 4.0% weight loss (Column (2)) but no significant change in height (Column (3)), suggesting that the decline in BMI is associated with the weight loss.^20^ From this we can further infer, as discussed earlier, that the lack of significant height shrinkage suggests that the BMI and weight estimates are less likely to have suffered from bias due to heterogeneous health shocks. In addition, there are no statistically significant changes in mental health conditions, suggesting that the results of BMI and weight are less likely to be suffering from bias due to the financial crisis of 2008.^21^

In contrast to university graduates, non-graduates show no statistically significant changes in any of the body measurements (Columns (4), (5), and (6)), indicating that health improvement with weight loss is observed only among university graduates at higher risk of obesity.^22^ From this, it appears that the policy reform was effective only for individuals with a higher level of education.

To round out the analysis, for individuals at lower risk of obesity before the policy reform, we found no statistically significant changes in any of the body measurements (Table 12) for either university graduates or non-graduates. This is not surprising, as individuals who are at lower risk of obesity are less likely to have an incentive to change their health condition because they are already healthy, at least by this measure.

### Effect on health investment behaviors

**Table 3:** Effects on health behaviors (University graduates with high pre-obesity risk)

				Eating habits					
	(1)	(2)	(3)	(4)	(5)	(6)	(7)	(8)	(9)
	Walking and exercise(HD)	Distance btw energy requirement and actual intake	= 1 if drinking alcohol	Staple food (g/d)	Main dishes (g/d)	Meat dishes (g/d)	Fish dishes (g/d)	Vegetables (g/d)	Ratio of fish dishes
Salaried before policy reform $\times$ after	0.277**	− 355.942**	− 0.223*	− 3.290	− 25.511	− 63.843**	38.332*	39.821	0.103***
	(0.124)	(159.630)	(0.119)	(88.964)	(31.167)	(24.629)	(21.731)	(46.141)	(0.037)
Number of observations	202	202	202	202	202	202	202	202	202
Control variables	Yes	Yes	Yes	Yes	Yes	Yes	Yes	Yes	Yes
Individual FE	Yes	Yes	Yes	Yes	Yes	Yes	Yes	Yes	Yes
Mean before the reform									
Salaried	0.14	512.91	0.83	475.64	437.05	270.86	166.19	254.08	0.38
Self-employed	0.06	620.66	0.79	501.78	431.66	263.10	168.56	247.36	0.38

*$$p<0.1$$, **$$p<0.05$$, ***$$p<0.01$$

All specifications are estimated using an FE model. Clustered robust standard errors are in parentheses

Included are age and marriage dummy variables, number of children, income, home ownership, hours worked, physical stress at the workplace, job stress at the workplace, current occupation dummy variables, cross terms of the occupation categories at 1st wave and survey year dummy variables, and prefecture level macroeconomic variables such as GDP and income per capita

This section discusses the estimation results for the effect of the reform on health investment behaviors related to obesity, focusing on physical activity, energy intake, drinking habits, and eating habits. Table 3 shows the estimation results for university graduates at higher pre-obesity risk. To measure physical activity, a dummy variable was constructed that takes a value of one if the respondent both walks 90 minutes on a normal day and takes part in some form of physical activity on holiday (Column (1)). I constructed the distance between the distance between actual and ideal levels of energy intake as a measure of energy intakes (Column (2)). I defined the ideal level as the estimated energy requirement (the level of energy intake required to maintain current body weight)(hereafter referred to EER) for individuals with low physical activity.^23^, ^24^ Column (3) reports estimates for a drinking dummy variable which takes value 1 if alcohol intake is greater than zero, and Columns (4) to (8) show the results for the daily intake of staple foods, main dishes, meat dishes, fish dishes, and vegetables, where main dish intake is the sum of meat and fish dishes intake. Column (9) shows the result for the ratio of fish dishes to main dishes intake.

From Table 3, the DID estimate for the physical activity dummy variable is positive and statistically significant, and the magnitude of the estimate can be interpreted as a 197 % increase in the amount of physical activity compared to the average value for salaried workers before the policy reform (Columns (1)). The DID estimate for the energy intake variable is negative and statistically significant, indicating that, for university graduates, the actual energy intake comes close to the ideal energy intake for weight loss (Columns (2)). The results suggest that, among university graduates, energy expenditure increases, and energy intake reaches the ideal level, which should result in weight loss. Further, among university graduates, salaried workers, compared to self-employed workers, stopped drinking after the policy reform at a significance level of 10 %, and the magnitude can be interpreted as a 26.87 % decline compared to the average before the policy reform (Column (3)). Salaried workers also statistically significantly changed their daily intake of meat and fish dishes, but not of staple foods, main dishes, or vegetables (Columns (4) to (8)). As the DID estimates for meat and fish dishes have opposite signs (meat is -63.843 and fish is 38.332) and the estimate for the ratio of fish to main dish intake is positive and statistically significant, this suggests that university graduates replaced meat with fish after the policy reform.^25^ In contrast, among non-graduates, no systematic changes in energy intake was observed while physical activity decreased (Table 13). Therefore, systematic changes in health behaviors were observed only among university graduates.

To sum up, we found that among university graduates at higher risk of obesity prior to the health policy reform, the revised health checkup resulted in changes in both health condition and health investment behaviors. BMI decreased to within the range associated with minimal all-cause mortality for middle aged Japanese men, and physical activity and eating habits improved. For all other groups, however, no change was observed in either health outcomes or behaviors.

## Discussion

### Interpretation of the DID estimates and comparison of university graduates and non-graduates

In the previous section, educational heterogeneity in the response to the policy reform is analyzed by comparing the DID estimates of university graduates and non-graduates. This section discusses three possible situations that complicate the comparison of the different effects between university graduates and non-graduates even when the common trend assumption between the treatment and control groups (salaried and self-employed workers) is satisfied. These are: (1) when the participation rate of salaried and self-employed workers is different, which is the case as this difference was important for our identification strategy, (2) when the health condition of salaried and self-employed workers is different prior to the reform, and (3) when employment status might be related to company size.

First, as discussed in “Identification strategy”, the DID estimate is interpreted as the lower bound of the magnitude of the effect of the policy reform in absolute value. Specifically, the estimate corresponds to the effect of the policy reform deducted by the difference in health checkup participation rates between salaried and self-employed workers. Therefore, it is difficult to compare the DID estimates for university graduates and non-graduates when the participation rates of salaried and self-employed workers among these two groups differ. For example, suppose that among university non-graduates the health checkup participation rate for salaried and self-employed workers is similar, so that the difference in participation rate is close to zero. Suppose, however, that there is a difference among university graduates. In such a situation, the DID estimate for non-graduates would be smaller in absolute value than for graduates even if the magnitude of the effect of the policy reform is the same for both the graduates and non-graduates. This occurs because there is no difference in the participation rate-which is the source of the difference in treatment intensity among the treatment and control groups-only for non-graduates.

**Table 4:** Differences in participation rate in health checkups by level of education

Panel A:	Participation rate		Difference
Comprehensive survey of living conditions	Salaried workers	Self-employed workers	
Whole sample	0.87	0.56	0.31***
University graduates$^1$	0.93	0.58	0.35***
Non-University graduates$^1$	0.85	0.57	0.29***

Panel B:	Participation rate		Difference
Longitudinal survey of middle-aged and elderly persons	Salaried workers	Self-employed workers	
Whole sample	0.87	0.51	0.35***
University graduates	0.91	0.53	0.38***
Non-University graduates	0.85	0.51	0.34***

*$$p<0.1$$, **$$p<0.05$$, ***$$p<0.01$$

${}^{1}$As the Comprehensive Survey of Living Conditions asks for level of education only after 2010, data for 2010, 2013, and 2016 are included

To investigate this possibility, Table 4 shows the difference in health checkup participation rates by level of education using two additional datasets: the Comprehensive Survey of Living Conditions (Panel A) and the Longitudinal Survey of Middle-aged and Elderly Persons (Panel B). The first and second columns show the participation rates of salaried and self-employed workers, and the third column shows the difference in their participation rates. The three rows of each panel show the results for the whole sample, that is, university graduates and non-graduates. Considering both panels, the difference in the participation rate ranges from 29 to 38 percentage points, with the difference for university graduates larger than for non-graduates (0.35 vs 0.29, and 0.38 vs 0.34), a single-digit percentage point difference. However, as seen in Table 2, the DID estimate of BMI for university graduates is several orders of magnitude larger (about 50 times larger) than for non-graduates (Column (1) vs Column (4)). Consequently, we can infer that the difference in the checkup participation rates of the two groups is not a major source of the difference in the estimation results.

A second concern in interpreting the DID estimates occurs when there is a difference in the pre-reform health conditions of salaried and self-employed workers because, as explained in “Institutional background”, the newly introduced system is comprised of two programs, health checkup and health guidance, and the specific intervention received depends on the participant’s assessed risk of metabolic syndrome. If salaried workers were less healthy than self-employed workers before the policy reform, they would have been more likely to receive health guidance than self-employed workers, causing the intensity of the effect of the policy reform to be larger among salaried workers. This would be problematic because it would conflate the DID estimates, with the difference in the DID estimates of university graduates and non-graduates perhaps only capturing the different health tendencies. For this reason, before conducting the estimation procedure, the sample was divided according to pre-reform health condition in order to address this issue.

**Fig. 4:**
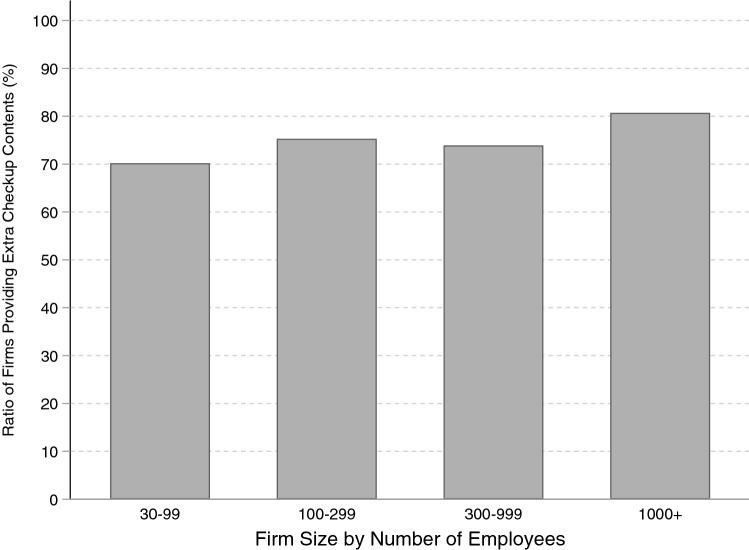
Relationship between firm size and provision of extra health checkup items. Source: General survey on working conditions 2007

**Fig. 5:**
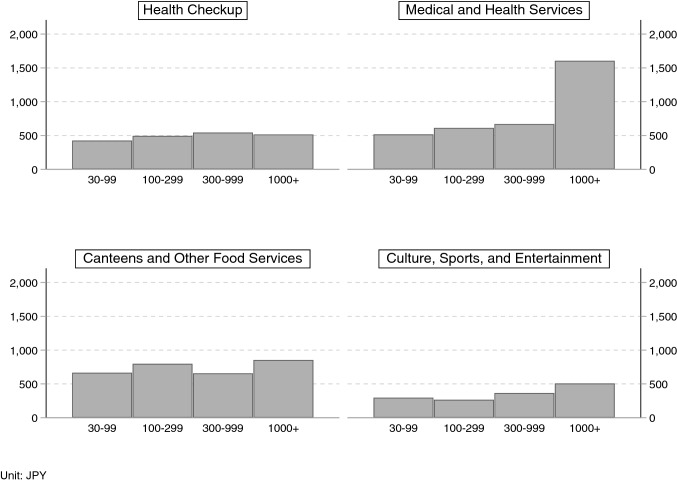
Relationship between firm size and benefit expenses not required by law. Source: General survey on working conditions 2011. Average monthly cost per capita

A third issue regarding the interpretation of the estimation results involves a possible conflation of employment status and workplace conditions. Recall that we found that only university graduates among salaried workers significantly changed their health conditions relative to self-employed workers. In Japan, salaried workers with university degrees are likely to work at large companies, which often provide more fringe benefits than smaller companies, and this could include extra health checkup items beyond those items required by law. If this is the case, then the significant effects observed for salaried university graduates might merely capture large company trends, thus we want to eliminate this possibility. According to the 2007 General Survey on Working Conditions, the ratio of firms providing extra health checkup items in addition to those required by law was almost the same regardless of firm size (Fig. 4). Similarly, the 2011 report shows that the cost to firms of fringe benefits not obligated by law related to health checkups is almost the same for all firm size categories (“health checkup” in Fig. 5). However, the cost of medical and health services for companies with over one thousand employees is approximately three times more than for smaller companies (“medical and health services” in Fig. 5).^26^ In order to address this issue, the model was estimated again including a firm size variable,^27^ and the results were found to be robust after this addition (Table 14).

To sum up, we found that the three potential issues raised in this section are not of concern, and so the DID estimates of the university graduates and non-graduates are comparable for discussing the heterogeneity of the response to the policy reform by level of education. The next section discusses possible reasons why the DID estimates are heterogeneous by level of education.

### Causes of heterogeneity by level of education and policy implications

This section discusses possible reasons for the observed heterogeneity by level of education in the responses to the health checkup, followed by policy implications. One possible explanation for the heterogeneous responses is a difference in cognitive functioning. In recent studies of the relation between education and health, there is much discussion about the relative contribution of variables at early stages of life, including cognitive and non-cognitive skills and socioeconomic background in childhood [4, 8]. According to Bijwaard et al. [4], at least half of the difference in the survival probability between educational groups can be explained by the selection of education choice, based mainly on cognitive skill. While Bijwaard and Van Kippersluis [3] finds that people with higher education are more efficient producers of health investment to survival probability than people with lower education; when cognitive skill is accounted for, the difference disappears.

In order to test this cognitive functioning hypothesis, the JSTAR memory and numeracy skill tests of cognitive functioning were used.^28^ Additionally, the discount rate, health preferences, and self-reported probability of being alive at age 80 are used to discuss other possible explanations of the heterogeneous response.^29^

**Table 5:** Differences in characteristics of salaried and self-employed workers by education

	University graduates	University Non-graduates	Difference
Cognitive Functioning Test Score:			
Word recall score (0–20)	11.03	10.21	0.82**
$\ge$ 3rd quartiles	0.48	0.33	0.16***
Serial 7s score (0–5)	4.57	4.34	0.23**
$\ge$ 3rd quartiles	0.74	0.63	0.11**
Discount rate	0.77	0.80	− 0.02
$\ge$ 3rd quartiles	0.44	0.44	0.00
Health preference:			
Interested in own health?			
Yes	0.88	0.86	0.02
No	0.01	0.04	− 0.03
Confident in own health?			
Yes	0.35	0.34	0.01
No	0.17	0.18	− 0.01
Body measurements:			
BMI	25.82	25.68	0.15
Self-reported probability			
of living at age 80	52.72	50.25	2.48
$\ge$ 3rd quartiles	0.29	0.31	− 0.01

*$$p<0.1$$, **$$p<0.05$$, ***$$p<0.01$$

First, according to Table 5, university graduates have statistically significantly higher cognitive functioning scores than non-graduates on the word recall test of memory functioning and the serial 7s numeracy test (8.0% and 5.3%, respectively). Moreover, the proportion of individuals whose word recall and serial 7s test scores are greater or equal to the 3rd quartile point is higher for university graduates than non-graduates (48.5% higher and 17.5% higher, respectively). In contrast, differences between university graduates and non-graduates on other characteristics such as the discount rate and self-reported probability of being alive at age 80 are not significant.

Next, in order to decompose the educational heterogeneity of the policy reform response into cognitive functioning and other factors, the university graduate and non-graduate samples were combined, and the model was estimated with the interaction terms of the DID term and variables, including university dummy variable, health preferences, cognitive functioning test score, discount rate, and self-rated probability of being alive at age 80. For this estimation, the latter three dummy variables took a value of one if the response was equal to or greater than the 3rd quartile point.

**Table 6:** Decomposition

	(1)	(2)	(3)	(4)	(5)	(6)
	BMI	Weight (kg)	Height (m)	BMI	Weight (kg)	Height (m)
Salaried before policy reform $\times$ after	− 0.020	0.177	0.002	1.314**	3.176*	− 0.004
	(0.291)	(0.748)	(0.002)	(0.622)	(1.636)	(0.003)
$\times$ Univ.=1	− 1.050**	− 3.253**	− 0.001	− 0.475	− 1.831	− 0.005
	(0.458)	(1.373)	(0.004)	(0.528)	(1.442)	(0.004)
$\times$ Serial7 score $\ge$ 3rd quartile				− 1.101**	− 2.650*	0.003
				(0.545)	(1.509)	(0.003)
$\times$ Word Recall score $\ge$ 3rd quartile				− 1.606***	− 4.380***	0.001
				(0.504)	(1.412)	(0.004)
$\times$ Discount Rate $\ge$ 3rd quartile				0.482	1.998	0.009**
				(0.570)	(1.662)	(0.004)
$\times$ Not Interested in own health				0.539	1.116	− 0.005
				(0.995)	(2.763)	(0.007)
$\times$ Not Confident in own health				− 0.550	− 1.256	0.004
				(0.638)	(1.975)	(0.005)
$\times$ Self-report prob. of living at 80 $\ge$ 3rd quartile				− 0.579	− 1.582	0.000
				(0.552)	(1.427)	(0.004)
Number of observation	638	638	638	515	515	515

*$$p<0.1$$, **$$p<0.05$$, ***$$p<0.01$$

All specifications are estimated using an FE model, with clustered robust standard errors in parentheses

Included are age and marriage dummy variables, number of children, income, home ownership, hours worked, physical stress at the workplace, job stress at the workplace, current occupation dummy variables, cross terms of the occupation categories at 1st wave and survey year dummy variables, the interaction term of the after dummy and firm size at the JSTAR 1st, and prefecture level macroeconomic variables such as GDP and income per capita, as well as the interaction terms between the control variables and the university dummy variable

Columns (1)–(3) of Table 6 show the results of the estimation without any interaction terms other than the university dummy variable. We see that the coefficients for the interaction of DID and university (“$\times$ Univ. = 1”) on BMI and weight are negative and statistically significant (Columns (1) and (2)), indicating that the DID estimates of BMI and weight are statistically significantly different between university graduates and non-graduates. However, after adding the other interaction terms, the coefficients for the interactions of DID and university on BMI and weight are no longer statistically significant (Columns (4) and (5)). Further, the coefficients for the interaction of DID and cognitive functioning test scores (“$\times$ Serial7 score $\ge$ 3rd quartile” and “$\times$ Word Recall score $\ge$ 3rd quartile”) on BMI and weight are statistically significantly negative while all other interaction terms are insignificant. This implies that cognitive functioning is one of the key factors explaining the educational heterogeneity of the responses to the health policy reform.^30^

This discussion concludes with a limitation of the estimation related to cognitive functioning test scores. Recall that the identification strategy relies on the difference in the participation rate between salaried and self-employed workers. As discussed above, if the difference in the participation rate were to differ between people with higher and lower cognitive functioning test scores, it would be difficult to interpret the coefficient of the DID and high cognitive test score interaction term. Because the Comprehensive Survey of Living Conditions and the Longitudinal Survey of Middle-aged and Elderly Persons do not include information about cognitive abilities, we were not able to conduct a secondary analysis to verify this. Therefore, we cannot unequivocally state that the coefficients of these interaction terms represent heterogeneity in the effects rather than heterogeneity in the participation rate of the health checkup.

## Conclusion

This study analyzed the effects of a policy reform of the health checkup system in Japan aimed at giving participants more objective information about their risk of obesity and health guidance from professionals on the health outcomes and health investment behaviors of participants, finding heterogeneity in the effects by level of education. According to the results of the estimation conducted using a DID framework, the estimates of BMI and weight were statistically significantly negative for university graduates at higher risk of obesity, indicating that the reform of the health checkup produced favorable health outcomes for these participants, who also improved their health investment behaviors including physical activity and eating habits. There were no significant changes observed, however, for non-graduates or even for graduates with relatively low risk of obesity either in terms of health outcomes or investment behaviors. The differences in the DID estimates between university graduates and non-graduates were statistically significant. These results suggest that highly educated people are more likely to respond to a health policy reform to change their behavior and improve their health condition.

In order to discover why this might be, a secondary analysis was conducted and revealed that cognitive functioning appears to be a key factor in explaining the educational heterogeneity of the responses to the policy reform. The descriptive statistics show that the university graduates in the analysis sample have statistically significantly higher cognitive functioning test scores than non-graduates, and a secondary analysis adding the interaction terms of DID and high cognitive test score dummies found these interaction terms to be statistically significantly negative. However, the difference in the DID estimates between university graduates and non-graduates was no longer significant, indicating that the reform was effective not strictly for university graduates but only for individuals with high cognitive functioning scores.

One possible mechanism is that it is easier for professionals such as doctors, public nurses, and dietitians to teach individuals with high cognitive functioning scores (i.e., they are more “teachable”). They may be more capable of efficiently processing the information from the checkup and subsequent guidance because of their higher cognitive functioning or because of more experience of being taught in their lives. In Specific Health Guidance, as discussed in “ Institutional background”, the participants are required to set their weight loss goals and make a lifestyle change plan to achieve them. They are also required to update their goals and plans as necessary. Thus, it is necessary for the participants to be able to process the information from the professionals, and teachability could be one of the key factors for the success of health guidance.

The policy implication is that interventions should be provided depending on individual characteristics, including not only pre-intervention health risk, but also teachability. For the interventions to be more successful, more intense interventions may be necessary for individuals who lack teachability. For the Japanese health checkup programs, a more effective health checkup with more wide-ranging effects could be provided through a more accessible presentation of health information and guidance that includes a clearly articulated explanation of the effects of specific identified risk factors and directly linking these risk factors to a concrete individualized action plan, especially for individuals who lack teachability. Health promotion policies that take individuals’ teachability into account could also have an important role in achieving health improvement for the entire population in other policy settings, and in other countries as well.

The limitations of the study that should be addressed in the future are as follows. First, this study holistically analyzed the effect of the policy reform of the Japanese checkup system which is now a two-part system of assessment and guidance, but it did not separate the relative effects of the assessment and guidance. Decomposing the reform effects into these two components is the subject of future work, as it may shed additional light on the observed educational heterogeneity of the reform. Second, as the first wave of the JSTAR only included five Japanese cities, and the sample size is relatively small, we need to pay attention to the external validity of the results. An expansion of this study to a more generalized population could also be the subject of future work. Third, although we tried to examine the validity of the common trend assumption by looking at self-reported health changes before the policy reform, using the observable characteristics as control variables, and implementing placebo regressions, we cannot confirm the parallel trend of the outcome variables between the treated and control groups, which is the typical way to discuss validation of the common trend assumption used in recent studies using the DID strategy. This is because, as explained in “Identification strategy”, we only have one period before the policy reform. Fourth, previous studies have argued that BMI is not a perfect measure of obesity. One reason is that BMI relies not only on fat, but also fat-free mass such as muscle and bone [5].^31^ Using other measures of obesity such as waist circumference and fat mass as outcome variables could be another subject of future work.

## Figures and Tables

**Fig. 6:**
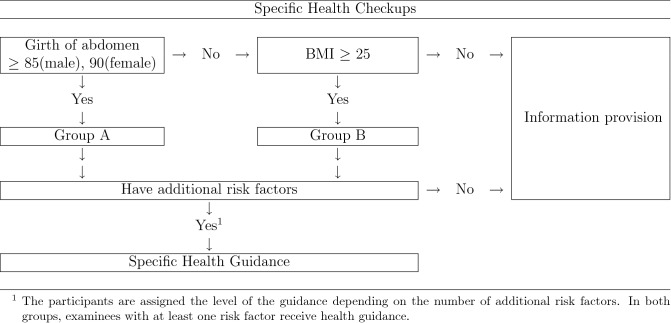
Screening procedure for health guidance. ${}^1$ The participants are assigned the level of the guidance depending on the number of additional risk factors. In both groups, examinees with at least one risk factor receive health guidance.

**Fig. 7:**
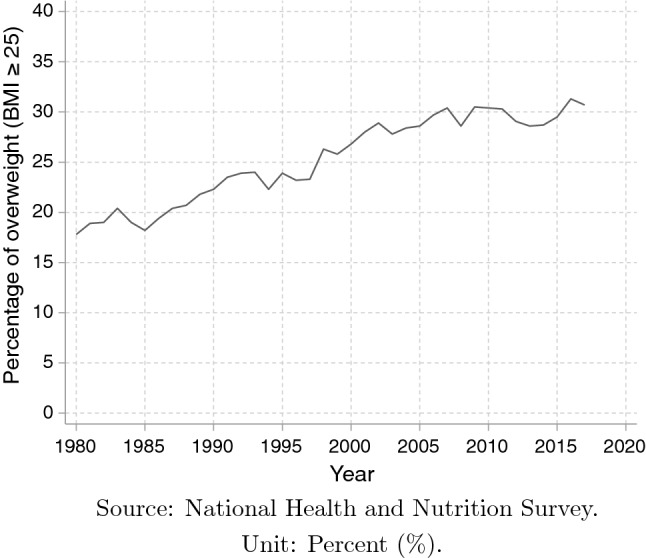
The change in percentage of overweight people (%). Source: National Health and Nutrition Survey. Unit: Percent (%)

**Fig. 8:**
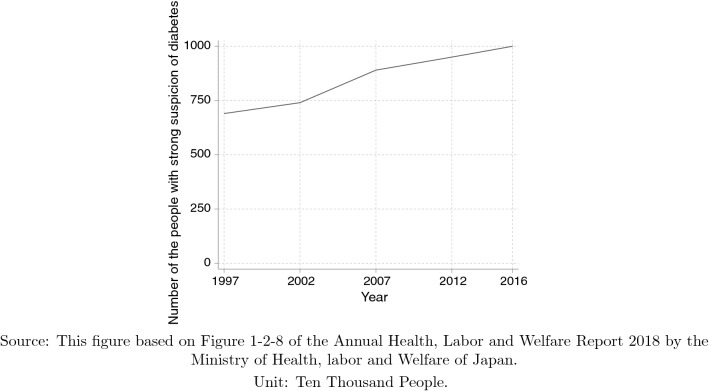
The change in the number of people strongly suspected of having diabetes (Ten thousand people). Source: This figure based on Figure 1-2-8 of the Annual Health, Labor and Welfare Report 2018 by the Ministry of Health, labor and Welfare of Japan. Unit: Ten Thousand People

## Screening procedure for the sSpecific health checkups

The screening process is divided into two parts: body measurement and additional risk factors. The procedure of the guidance status is as follows:

- First, examinees are divided by the girth of their abdomen, with examinees whose abdomen is over the criteria (male:85cm, female:90cm) assigned to group A.
- Second, those whose abdomen girth is below the criteria (not in group A) but with body mass index (BMI) above 25 are assigned to group B .
- Additionally, the risk level of examinees in group A or B are evaluated by four additional risk factors: high blood sugar, lipid abnormality, high blood pressure, and smoking history.^32^
- In group A, examinees with more than two risk factors receive active support guidance, examinees with one risk factor receive motivational support guidance, and examinees without any risk factors are provided with information about their health but do not receive any guidance.
- Similarly, in group B, examinees with more than three risk factors receive active support guidance, examinees with one or two risk factors receive motivational support guidance, and examinees without any risk factors are provided with information but not guidance.
- Examinees not in group A or B are provided with the information about their health but do not receive any guidance.

The procedure is summarized in Fig. 6. If participants have at least one additional risk factor, then they are categorized as people with high risk. Here participants are able to update their precise risk of metabolic syndrome onset.

## Additional descriptive statistics and estimation results

Figures 7 and 8 show the change in the percentage of overweight people and the change in the number of the people strongly suspected of having diabetes, respectively.

**Table 7:** Effects of policy reform on body measurements by education and pre-BMI category (male aged 50–62 with high pre-obesity risk)

	University graduates			University non-graduates		
	(1)	(2)	(3)	(4)	(5)	(6)
	BMI	Weight (kg)	height (m)	BMI	Weight (kg)	height (m)
Salaried before policy reform $\times$ After (DID)	− 0.404	− 1.048	0.003	0.240	0.676	0.000
	(0.425)	(1.267)	(0.004)	(0.400)	(1.013)	(0.003)
$\times$ 26.0 $\le$ pre-reform BMI < 28.5	− 1.762*	− 5.334*	− 0.007	− 0.682	− 1.301	0.007
	(1.009)	(2.985)	(0.005)	(0.609)	(1.569)	(0.007)
$\times$ pre-reform BMI $\ge$ 28.5	− 4.771***	− 14.804***	− 0.005	− 1.380	− 3.379	0.004
	(0.901)	(2.608)	(0.008)	(0.869)	(2.456)	(0.007)
Number of observations	202	202	202	480	480	480
Mean before the policy reform for salaried workers by pre-reform BMI						
23.5 $\le$ pre-BMI < 26.0	24.58	70.73	1.70	24.63	69.39	1.68
26.0 $\le$ pre-BMI < 28.5	27.02	78.89	1.71	27.06	75.94	1.67
Pre-BMI $\ge$ 28.5	30.11	86.60	1.70	29.96	83.06	1.66
${\hat{\beta }}_{DID}+{\hat{\beta }}_{{DID \times 26.0 \le pre-reform \, BMI< 28.5}}$	− 2.166	− 6.382	− 0.004	− 0.442	− 0.624	0.007
$p-value$	0.013	0.014	0.381	0.340	0.604	0.257
${\hat{\beta }}_{DID}+{\hat{\beta }}_{{DID \times pre-reform \,BMI\ge 28.5}}$	− 5.175	− 15.853	− 0.002	− 1.139	− 2.702	0.004
$p-value$	0.000	0.000	0.758	0.144	0.230	0.476

*$$p<.1$$, **$$p<.05$$, ***$$p<.01$$

All specifications are estimated using an FE model, with clustered robust standard errors in parentheses

Included are age and marriage dummy variables, number of children, income, home ownership, hours worked, physical stress at the workplace, job stress at the workplace, current occupation dummy variables, cross terms of the occupation categories at 1st wave and survey year dummy variables, and prefecture level macroeconomic variables such as GDP and income per capita. The models also include the first- and second-order terms of the triple cross terms of the DID and pre-reform BMI categories. The reference term of the pre-BMI categories is the category, “23.5 $\le$ pre-reform BMI < 26.0.”

We also report the estimates of the sum of the coefficients of DID and those of DID $\times$ pre-BMI categories (${\hat{\beta }}_{DID}+{\hat{\beta }}_{{DID \times 26.0 \le pre-reform \ BMI< 28.5}}$ and ${\hat{\beta }}_{DID}+{\hat{\beta }}_{{DID \times pre-reform \ BMI\ge 28.5}}$), which corresponds to the effect of policy reform on individuals with each pre-reform BMI category, and the *p* value of a test statistically confirming whether the sum is zero ($p value$)

Table 7 summarizes the estimation results of the policy reform effects on body measurements by education and pre-reform BMI category for males aged 50 to 62 with high pre-obesity risk. We added the triple cross terms of the DID term and pre-reform BMI categories into Equation (1), as a triple difference model. We divide the pre-reform BMI value into three categories: “23.5 $\le$ pre-reform BMI < 26.0,” “26.0 $\le$ pre-reform BMI < 28.5,” and “pre-reform BMI $\ge$ 28.5.” The category, “23.5 $\le$ pre-reform BMI < 26.0,” is used as the reference category in the estimation. We report the DID estimate and the estimates of the cross term of the DID term and pre-reform BMI categories. We also report the estimates of the sum of the coefficients of DID and those of DID $\times$ pre-BMI categories (${\hat{\beta }}_{DID}+{\hat{\beta }}_{{DID \times 26.0 \le pre-reform \ BMI< 28.5}}$ and ${\hat{\beta }}_{DID}+{\hat{\beta }}_{{DID \times pre-reform \ BMI\ge 28.5}}$), which corresponds to the effect of policy reform on individuals with each pre-reform BMI category, and the *p* value of a test statistically confirming whether the sum is zero ($p value$).

According to Table 7, among the university graduates, the DID estimate for BMI itself is negative but statistically insignificant (Column (1)), unlike Table 2. The average pre-reform BMI for the treated group among the university graduates in the lowest category is relatively low at 24.58, which is in the BMI range that minimizes all-cause mortality among middle-aged Japanese, thus the result is not so surprising. The estimates of the cross term of the DID term and pre-reform BMI categories for BMI are negative and statistically significant, and the estimates of the sum of the coefficients of DID and those of DID $\times$ pre-BMI categories are also negative and statistically significant. Compared to the average BMI for salaried workers with a university degree before the policy reform, the effect of policy reform on individuals with each pre-reform BMI category can be interpreted as 8.02 %, and 17.19 % decreases in BMI for the categories, “26.0 $\le$ pre-reform BMI < 28.5” and “pre-reform BMI $\ge$ 28.5,” respectively. The result suggests that the higher the pre-reform BMI, the larger the magnitude of BMI reduction due to the policy reform. The same tendency is observed for the results of university graduates’ weight loss (Column (2)).

**Table 8:** Effects of policy reform on mental health condition by education (male aged 50–62 with high pre-obesity risk)

	University graduates		University non-graduates	
	(1)	(2)	(3)	(4)
	CESD (0-60)	CESD $\ge$ 16	CESD (0-60)	CESD $\ge$ 16
Salaried before policy reform $\times$ after	− 2.008	− 0.144	1.529	− 0.037
	(1.894)	(0.191)	(1.564)	(0.127)
Number of observation	202	202	475	475
Control variables	Yes	Yes	Yes	Yes
Individual FE	Yes	Yes	Yes	Yes
Mean before the reform				
Salaried	11.18	0.13	11.93	0.18
Self-employed	10.68	0.12	11.92	0.11

*$$p<0.1$$, **$$p<0.05$$, ***$$p<0.01$$

All specifications are estimated using an FE model, with clustered robust standard errors in parentheses

Included are age and marriage dummy variables, number of children, income, home ownership, hours worked, physical stress at the workplace, job stress at the workplace, current occupation dummy variables, cross terms of the occupation categories at 1st wave and survey year dummy variables, and prefecture level macroeconomic variables such as GDP and income per capita

Table 8 in Appendix B summarizes the estimation results for males aged 50-62 with higher pre-obesity risk. There are no statistically significant impacts of the policy reform on mental health conditions.

**Table 9:** Robustness check for age range (BMI)

	University graduates			University non-graduates		
	(1)	(2)	(3)	(4)	(5)	(6)
	Age 50–62	Age 50–61	Age 50-60	Age 50–62	Age 50–61	Age 50-60
Salaried before policy reform $\times$ after	− 1.065***	− 1.092***	− 1.226***	− 0.021	0.066	0.188
	(0.321)	(0.328)	(0.371)	(0.287)	(0.303)	(0.334)
Number of observations	202	184	170	480	419	365
Control variables	Yes	Yes	Yes	Yes	Yes	Yes
Individual FE	Yes	Yes	Yes	Yes	Yes	Yes
Mean before the reform						
Salaried	25.78	25.77	25.80	25.68	25.63	25.65
Self-employed	25.93	25.97	26.02	25.66	25.74	25.76

*$$p<0.1$$, **$$p<0.05$$, ***$$p<0.01$$

All specifications are estimated using an FE model, with clustered robust standard errors in parentheses.

Included are age and marriage dummy variables, number of children, income, home ownership, hours worked, physical stress at the workplace, job stress at the workplace, current occupation dummy variables, cross terms of the occupation categories at 1st wave and survey year dummy variables, and prefecture level macroeconomic variables such as GDP and income per capita

**Table 10:** Robustness check for age range (Weight)

	University graduates			University non-graduates		
	(1)	(2)	(3)	(4)	(5)	(6)
	Age 50–62	Age 50–61	Age 50–60	Age 50–62	Age 50–61	Age 50-60
Salaried before policy reform $\times$ after	− 2.987***	− 3.022***	− 3.686***	0.166	0.411	0.667
	(1.071)	(1.061)	(1.223)	(0.740)	(0.791)	(0.884)
Number of observations	202	184	170	480	419	365
Control variables	Yes	Yes	Yes	Yes	Yes	Yes
Individual FE	Yes	Yes	Yes	Yes	Yes	Yes
Mean before the reform						
Salaried	74.41	74.48	74.58	72.16	72.11	72.32
Self-employed	72.59	72.88	73.10	71.67	72.02	72.20

*$$p<0.1$$, **$$p<0.05$$, ***$$p<0.01$$

All specifications are estimated using an FE model, with clustered robust standard errors in parentheses

Included are age and marriage dummy variables, number of children, income, home ownership, hours worked, physical stress at the workplace, job stress at the workplace, current occupation dummy variables, cross terms of the occupation categories at 1st wave and survey year dummy variables, and prefecture level macroeconomic variables such as GDP and income per capita

**Table 11:** Robustness check for age range (Height)

	University graduates			University non-graduates		
	(1)	(2)	(3)	(4)	(5)	(6)
	Age 50–62	Age 50-61	Age 50–60	Age 50–62	Age 50–61	Age 50–60
Salaried before policy reform $\times$ after	0.002	0.002	− 0.001	0.003	0.003	0.002
	(0.004)	(0.004)	(0.004)	(0.002)	(0.003)	(0.003)
Number of observations	202	184	170	480	419	365
Control variables	Yes	Yes	Yes	Yes	Yes	Yes
Individual FE	Yes	Yes	Yes	Yes	Yes	Yes
Mean before the reform						
Salaried	1.70	1.70	1.70	1.68	1.68	1.68
Self-employed	1.67	1.67	1.68	1.67	1.67	1.67

*$$p<.1$$, **$$p<.05$$, ***$$p<.01$$

All specifications are estimated using an FE model, with clustered robust standard errors in parentheses

Included are age and marriage dummy variables, number of children, income, home ownership, hours worked, physical stress at the workplace, job stress at the workplace, current occupation dummy variables, cross terms of the occupation categories at 1st wave and survey year dummy variables, and prefecture level macroeconomic variables such as GDP and income per capita

Tables 9, 10, and 11 summarize the estimation results for robustness checks against age ranges. The estimation results are robust for other age ranges as well.

**Table 12:** Effects of policy reform on body measurements by education (males aged 50–62 with low pre-obesity risk)

	University graduates			University non-graduates		
	(1)	(2)	(3)	(4)	(5)	(6)
	BMI	Weight (kg)	Height (m)	BMI	Weight (kg)	Height (m)
Salaried before policy reform $\times$ after	0.262	1.378	0.008	0.168	0.422	-0.000
	(0.416)	(1.138)	(0.005)	(0.221)	(0.658)	(0.003)
Number of observations	214	214	214	556	556	556
Control variables	Yes	Yes	Yes	Yes	Yes	Yes
Individual FE	Yes	Yes	Yes	Yes	Yes	Yes
Mean before the reform						
Salaried	21.29	60.53	1.69	21.41	59.84	1.67
Self-employed	21.14	60.13	1.69	21.56	59.64	1.66

*$$p<0.1$$, **$$p<0.05$$, ***$$p<0.01$$

All specifications are estimated using an FE model, with clustered robust standard errors in parentheses

Included are age and marriage dummy variables, number of children, income, home ownership, hours worked, physical stress at the workplace, job stress at the workplace, current occupation dummy variables, cross terms of the occupation categories at 1st wave and survey year dummy variables, and prefecture level macroeconomic variables such as GDP and income per capita

Table 12 shows the estimation results for those at lower risk of obesity before the policy reform by level of education, with Columns (1), (2), and (3) showing university graduates and Columns (4), (5), and (6) showing non-graduates. For individuals at lower risk of obesity before the policy reform, we found no statistically significant changes in any of the body measurements for either university graduates or non-graduates.

**Table 13:** Effects on health behaviors (University non-graduates with high pre-obesity risk)

				Eating Habits					
	(1)	(2)	(3)	(4)	(5)	(6)	(7)	(8)	(9)
	Walking and exercise(HD)	Distance btw energy requirement and actual intake	= 1 if drinking alcohol	Staple food (g/d)	Main dishes (g/d)	Meat dishes (g/d)	Fish dishes (g/d)	Vegetables (g/d)	Ratio of fish dishes
Salaried before policy reform $\times$ after	− 0.168**	151.719	− 0.029	36.372	− 7.920	7.666	− 15.586	8.381	− 0.026
	(0.075)	(93.697)	(0.085)	(46.294)	(27.762)	(20.930)	(14.253)	(23.870)	(0.025)
Number of observations	480	480	480	480	480	480	480	480	479
Control variables	Yes	Yes	Yes	Yes	Yes	Yes	Yes	Yes	Yes
Individual FE	Yes	Yes	Yes	Yes	Yes	Yes	Yes	Yes	Yes
Mean before the reform									
Salaried	0.12	498.12	0.77	493.38	425.18	255.87	169.30	243.96	0.40
Self-employed	0.10	493.29	0.70	545.23	441.00	282.81	158.19	250.42	0.36

*$$p<0.1$$, **$$p<0.05$$, ***$$p<0.01$$

All specifications are estimated using an FE model, with clustered robust standard errors in parentheses.

Included are age and marriage dummy variables, number of children, income, home ownership, hours worked, physical stress at the workplace, job stress at the workplace, current occupation dummy variables, cross terms of the occupation categories at 1st wave and survey year dummy variables, and prefecture level macroeconomic variables such as GDP and income per capita

Table 13 summarizes the estimation results of the analysis on health behaviors for non-graduates with higher pre-obesity risk. Among non-graduates, no systematic change in energy intake and eating habits was observed while physical activity decreased.

**Table 14:** Robustness check for firm size effect

	University graduates			University non-graduates		
	(1)	(2)	(3)	(4)	(5)	(6)
	BMI	Weight (kg)	Height (m)	BMI	Weight (kg)	Height (m)
Panel A: without firm size variable (Table 2)						
Salaried before policy reform $\times$ after	− 1.065***	− 2.987***	0.002	− 0.021	0.166	0.003
	(0.321)	(1.071)	(0.004)	(0.287)	(0.740)	(0.002)
Number of observations	202	202	202	480	480	480
Panel B: with firm size variable						
Salaried before policy reform $\times$ after	− 1.069***	− 3.076**	0.001	− 0.020	0.177	0.002
	(0.371)	(1.209)	(0.003)	(0.286)	(0.735)	(0.002)
Number of observations	197	197	197	441	441	441

*$$p<0.1$$, **$$p<0.05$$, ***$$p<0.01$$

All specifications are estimated using an FE model, with clustered robust standard errors in parentheses.

Included are age and marriage dummy variables, number of children, income, home ownership, hours worked, physical stress at the workplace, job stress at the workplace, current occupation dummy variables, cross terms of the occupation categories at 1st wave and survey year dummy variables, and prefecture level macroeconomic variables such as GDP and income per capita

Table 14 summarizes the estimation results for the robustness check for the firm size variable. The results were found to be robust after adding the firm size variable to the estimation model.

**Table 15:** Decomposition (Health Behaviors)

				Eating Habits					
	(1)	(2)	(3)	(4)	(5)	(6)	(7)	(8)	(9)
	Walking and exercise(HD)	Distance btw energy requirement and actual intake	= 1 if drinking alcohol	Staple food (g/d)	Main dishes (g/d)	Meat dishes (g/d)	Fish dishes (g/d)	Vegetables (g/d)	Ratio of fish dishes
Salaried before policy reform $\times$ after	− 0.340*	346.338*	0.020	− 44.387	− 75.148	-30.572	− 44.575	− 29.831	− 0.008
	(0.176)	(188.711)	(0.220)	(78.714)	(48.747)	(39.540)	(27.194)	(52.078)	(0.055)
$\times$ Univ.=1	0.605***	-264.963	− 0.317*	− 146.842	− 30.906	− 69.306*	38.399	15.749	0.099**
	(0.154)	(193.351)	(0.187)	(99.745)	(57.599)	(41.343)	(30.539)	(68.767)	(0.049)
$\times$ Serial7 score $\ge$ 3rd quartile	0.255	− 141.547	0.066	76.138	4.984	− 28.140	33.124	50.198	0.049
	(0.168)	(189.680)	(0.195)	(96.858)	(52.975)	(40.322)	(28.954)	(52.395)	(0.054)
$\times$ Word Recall score $\ge$ 3rd quartile	− 0.097	− 258.668	− 0.003	− 121.411	70.490	57.308	13.183	2.566	− 0.036
	(0.155)	(182.529)	(0.201)	(83.779)	(55.855)	(41.652)	(27.354)	(53.702)	(0.046)
$\times$ Discount Rate $\ge$ 3rd quartile	− 0.050	− 172.280	− 0.045	130.728	33.635	25.630	8.005	38.954	0.014
	(0.162)	(183.249)	(0.154)	(94.972)	(63.571)	(50.106)	(25.428)	(58.985)	(0.050)
$\times$ Be not intrested in own health	− 0.192	762.919***	− 0.440	377.059**	96.863	66.764	30.099	55.124	0.020
	(0.236)	(258.496)	(0.305)	(157.371)	(130.020)	(90.535)	(57.001)	(155.656)	(0.086)
$\times$ Do not have confidence in own health	− 0.272	− 204.711	0.078	− 83.045	93.269	58.127	35.141	− 45.559	-0.063
	(0.225)	(232.663)	(0.171)	(111.396)	(71.347)	(54.920)	(34.478)	(72.632)	(0.064)
$\times$ Self-rated probability of living at age 80 $\ge$ 3rd quartile	0.281*	165.991	− 0.198	− 59.153	28.946	23.877	5.069	9.383	-0.032
	(0.150)	(193.674)	(0.136)	(80.628)	(61.075)	(47.926)	(28.205)	(53.612)	(0.052)
Number of observations	515	515	515	515	515	515	515	515	515
Control variables	Yes	Yes	Yes	Yes	Yes	Yes	Yes	Yes	Yes
Individual FE	Yes	Yes	Yes	Yes	Yes	Yes	Yes	Yes	Yes
Mean before the reform									
Salaried	0.13	502.43	0.79	488.21	428.63	260.24	168.40	246.90	0.40
Self-employed	0.08	533.76	0.73	531.42	438.03	276.55	161.48	249.45	0.37

*$$p<0.1$$, **$$p<0.05$$, ***$$p<.01$$

All specifications are estimated using an FE model, with clustered robust standard errors in parentheses

Included are age and marriage dummy variables, number of children, income, home ownership, hours worked, physical stress at the workplace, job stress at the workplace, current occupation dummy variables, cross terms of the occupation categories at 1st wave and survey year dummy variables, and prefecture level macroeconomic variables such as GDP and income per capita, as well as interaction terms between the control variables and the university dummy variable

Table 15 summarizes the estimation results on health behaviors for decomposing the educational heterogeneity of the policy reform response into cognitive functioning and other factors. No systematic tendencies were observed in the estimation.

## Footnotes

1 These two mechanisms are similar to the mechanisms discussed in Price and Simon [35].

2 They found no evidence for biomarkers, such as blood pressure, hemoglobin A$_{1c}$ level, or low-density lipoprotein cholesterol level.

3 In general, participants of health checkup and screening programs receive not only a diagnosis of diseases but also a value of biomarkers. If participants know a biomarker threshold of a disease and know that their values of the biomarker are just below the threshold, they may change behaviors to improve their health conditions because they know that they are at high risk of the disease. In this case, the effect estimated by the RDD with the biomarker threshold should be the lower bound of the magnitude of the effect in absolute value. While the results are conservative, we cannot identify whether the effects on the insignificant results are either null or being attenuated by the strategy.

4 Additionally, the authors note that the sample size of the highly educated group is relatively small, which may have led to imprecise estimates.

5 Figures 7 and 8 show the change in the percentage of overweight people and the change in the number of people strongly suspected of having diabetes, respectively.

6 Further information (in Japanese) can be found at: https://www.wam.go.jp/wamappl/bb14GS50.nsf/vAdmPBigcategory40/98E6F3F836572E8B4925716F0006B833?OpenDocument and https://www.mhlw.go.jp/shingi/2005/09/s0915-8.html.

7 The Schellenberg et al. [37] systematic review of diabetes, for example, found no evidence that intervention is effective for those who already have type 2 diabetes but is effective for those at high risk of getting it.

8 In motivational support guidance, first, the participants receive an individual or group face-to-face consultation with professionals such as doctors, public nurses, or dietitians. The professionals explain the relationship between participants’ lifestyles and the health risks revealed by the health checkup, and the potential consequences of ignoring the identified risk factors. They also provide support to set participants’ goals of weight loss and to plan lifestyle changes to achieve the goals. Six months after the initial consultation, the professionals then evaluate the progress of participants’ weight loss face-to-face, via telephone, or via email. Note that in order to ensure continuity of the support, in principle, the same person who conducted the initial consultation conducts the six-months-after evaluation. In active support guidance, participants receive the initial consultation and the evaluation six months after the initial consultation, similar to motivational support guidance. The difference between motivational and active support guidance is that, in active support guidance, participants can receive continuous support from professionals for more than three months between the initial consultation and six-months-after evaluation. The continuous support for over three months includes a mid-term evaluation of weight loss progress, updating the lifestyle improvement plan as needed, and encouraging participants to stick with the plan, and this is done face-to-face, via telephone, or via emails. Each support system is assigned a score, and the professionals construct a combination of supports such that the sum of the score exceeds certain criteria.

9 The cost of health checkups and health guidance is either free or very low for the participants. For salaried workers, companies bear the cost of annual health checkups. For self-employed workers, local governments bear most of the cost. In all five cities in the JSTAR, the out-of-pocket expense for health checkups is free or below 1,000 JPY.

10 Please see Ichimura et al. [20] and http://www.rieti.go.jp/en/projects/jstar/index.html for more detailed information about the JSTAR.

11 There is a difference in the type of health insurance between salaried and self-employed workers. In Japan, salaried workers have employees’ health insurance (EHI) where both employees and employers pay insurance fees, and self-employed workers have national health insurance (NHI) mainly operated by local governments. However, there is no large difference between the EHI and NHI. First, the participation rate of health insurance does not differ between the EHI and NHI because Japan has a universal health care insurance system, that is, almost all residents of Japan have health insurance. Second, health care services covered by health insurance are the same between the EHI and NHI because the Japanese government decides the coverage and price of health care services covered by health insurance uniformly. Third, in Japan, people can freely choose medical institutions when they want to see a doctor. So, choice sets of medical institutions are not different between the EHI and NHI. We discuss the other aspects of the differences between salaried and self-employed workers in Table 1 and summarize the difference in the observable characteristics between the two groups in “Estimation results”.

12 Non-working individuals who were retired or disabled in 2007 are not included in the control group because they may have different trends compared to those who were working.

13 We use two types of occupation variables. One in the current occupation dummies. The occupation is classified as follows: “specialist and technical workers,” “administrative and managerial workers,” “clerical workers,” “sales workers,” “service workers,” “security workers,” “agriculture, forestry, and fishery workers,” “transport and communication workers,” and “production process and related workers.” The other is the cross terms of the occupation category variables at 1st wave and survey year dummies. The occupation category at 1st wave is classified as follows: office workers (“specialist and technical workers,” “administrative and managerial workers,” and “clerical workers,”), factory workers (“production process and related workers”), and other workers (“sales workers,” “service workers,” “security workers,” “agriculture, forestry, and fishery workers,” “transport and communication workers”).

14 The 2007 JSTAR asks the following question: “How is your current health compared to one year ago?” (question number: D-003) The options are “much better,” “better,” “same’,’ “worse,” “much worse.” In the figure, “better” includes both “much better” and “better” responses to the original question, while “worse” includes both “much worse” and “worse.”

15 For comparison, in England, an annual rate of height loss is estimated between 0.08 % and 0.10 % for males over 50 years old using the ELSA [12].

16 Assuming that body weight for treatment and control groups are the same, 75 kg, and that height declines by 1.02 cm from 170 cm only among the control group. Here, the value 1.02 cm is assumed by multiplying the six years (years from the first wave of the JSTAR to the fourth wave) and the upper bound of the estimated annual height decline by Otsuka et al. [34], 0.17. In this instance, the control group’s BMI value increases by 0.31 while the treatment group’s does not change. This produces a DID estimate implying a BMI reduction without weight loss among the treatment group.

17 We excluded observations of BMI values that are less than the one percentile and more than the 99 percentile of the BMI distribution from the analysis sample because these BMI values could be outliers. The one and the 99 percentiles are 16.7969 and 31.5963, respectively.

18 The evaluation derived here is only an approximation because of a lack of information about blood glucose and triglyceride levels which are also risk factors.

19 Table 7 summarizes the estimation results of the policy reform effects on body measurements by education and pre-reform BMI category for males aged 50–62 with high pre-obesity risk. Appendix B discusses the estimation results further.

20 The effect of the policy reform on weight is also non-linear in the same way as the effect on BMI (Column (2) of Table 7).

21 We use the Center for Epidemiologic Studies Depression Scale (CES-D) as the measurement of mental health conditions. A higher CES-D score indicates more adverse mental health conditions. We use both the continuous value of the CES-D scores and the dummy, indicating that the CES-D scores are greater than or equal to 16 as dependent variables. The value of 16 is used as a cutoff point for the diagnosis of a mental problem. Table 8 in Appendix B summarizes the estimation results for males aged 50–62 at higher pre-obesity risk.

22 The estimation results are robust for other age ranges as well (Tables 9, 10, and 11).

23 According to Sasaki [36], EER is determined by multiplying the basal metabolic rate (BMR) and the physical activity level (PAL). BMR was calculated using the prediction equations developed by Ganpule et al. [14], with $BMR = 0.1238 + 0.0481 \times Weight(kg) + 0.0234 \times Height(cm)$-$~ 0.0138 \times Age$- 0.5473 for males. Based on “Reference 1” of Sasaki [36], a PAL value of 1.5 was chosen, corresponding to the level for a sedentary lifestyle, and actual energy intake was obtained from the JSTAR nutrition survey.

24 To achieve weight loss, actual energy intake should be lower than the EER for one’s current lifestyle, so if one takes part in a moderate level of physical activity, actual energy intake should be lower than the EER for the moderate level. “Reference 1” of Sasaki [36] classifies sedentary workers with some movement as moderately physically active and workers with physically demanding positions as highly physically active. On this basis, the analysis sample for the current study includes only workers whose level of physical activity is moderate or above. If the analysis sample’s energy intake is close to the EER for the low level of physical activity, they should lose weight. So, we used the EER for the low level of physical activity as the ideal level of energy intake for weight loss.

25 Compared to the average before the policy reform, the magnitude of the DID estimate can be interpreted as a 23.57 % decline and a 23.07 % increase for meat and fish dishes, respectively. In the same manner, the effect for the ratio of fish to main dish intake can be interpreted as a 27.11 % increase.

26 In Japan, a company with over seven hundred employees can create a health insurance society for their employees. In the General Survey on Working Conditions, if a firm has a health insurance society, the firm’s cost for medical and health services can include the society’s labor administration costs. Therefore, it is possible that any difference in the cost for medical and health services observed between large and smaller companies may at least partially be explained by the cost of administering the health insurance society rather than any difference in direct support for employees.

27 The interaction term of the after dummy and firm size from the JSTAR 1st wave was chosen as the firm size variable to capture firm size trends.

28 We used the cognitive functioning test scores from the first wave of the JSTAR. For respondents with missing test scores from the first JSTAR, we used those from the second wave instead.

29 The values from the first wave of JSTAR were used.

30 In addition to health outcomes, the model was also estimated for health investment behaviors, but no systematic tendencies were observed (Table 15).

31 Johansson et al. [23] also discussed the reliability of BMI as an obesity measure in the economic literature.
